# Iron–Sulfur Cluster Biogenesis as a Critical Target in Cancer

**DOI:** 10.3390/antiox10091458

**Published:** 2021-09-14

**Authors:** Michael S. Petronek, Douglas R. Spitz, Bryan G. Allen

**Affiliations:** 1 Department of Radiation Oncology, Division of Free Radical and Radiation Biology, The University of Iowa Hospitals and Clinics, Iowa City, IA 52242-1181, USA; douglas-spitz@uiowa.edu; 2 Holden Comprehensive Cancer Center, Free Radical and Radiation Biology Program, Department of Radiation Oncology, University of Iowa, Iowa City, IA 52242-1181, USA

**Keywords:** iron–sulfur cluster biogenesis, iron metabolism, carcinogenesis, cancer therapy

## Abstract

Cancer cells preferentially accumulate iron (Fe) relative to non-malignant cells; however, the underlying rationale remains elusive. Iron–sulfur (Fe–S) clusters are critical cofactors that aid in a wide variety of cellular functions (e.g., DNA metabolism and electron transport). In this article, we theorize that a differential need for Fe–S biogenesis in tumor versus non-malignant cells underlies the Fe-dependent cell growth demand of cancer cells to promote cell division and survival by promoting genomic stability via Fe–S containing DNA metabolic enzymes. In this review, we outline the complex Fe–S biogenesis process and its potential upregulation in cancer. We also discuss three therapeutic strategies to target Fe–S biogenesis: (i) redox manipulation, (ii) Fe chelation, and (iii) Fe mimicry.

## 1. Introduction

There is growing evidence that cancer cells preferentially take up and sequester iron (Fe) relative to non-malignant cells, and it has recently been hypothesized that Fe is a central connection between the genetic and metabolic theories of cancer [1]. Transferrin receptors (TfR) are frequently upregulated on cancer cells, indicating a potential for increased Fe flux [2]. TfR expression is transcriptionally regulated by the transcription factors c-Myc and HIF-1α [3,4]. C-Myc and HIF-1α expression are associated with tumor aggressiveness [3,4]. Conversely, cancer cells can limit iron export by producing hepcidin to promote ferroportin (Fpn-1) degradation [5]. Both the cause and impact of this cancer cell iron-dependent phenotype have not been well characterized.

Fe is an essential cofactor in many processes that are key features of cancer [1]. Loosely bound, redox-active (commonly referred to as “labile”) iron has garnered significant interest due to its ability to catalyze the formation of reactive oxygen species and induce DNA damage via Fenton chemistry [6,7]. The redox-active iron pool (RIP) inside the cell is small, comprising ≤ 5% of the total iron content [8,9]. Despite data suggesting an increased RIP in cancer cells, the percentage change between malignant and non-malignant cells is minimal [10]. This suggests that most of the intracellular iron is used for functional purposes. 

Intracellular Fe may be utilized in three forms: (1) Fe–S biogenesis, (2) heme synthesis, and (3) mono- and di-iron proteins [11]. Both Fe–S biogenesis and heme synthesis occur on the inner mitochondrial membrane [12]. Heme synthesis may be regulated by Fe–S biogenesis as the terminal enzyme of the heme synthesis pathway, ferrochelatase (FECH), which contains a [2Fe-2S] cluster that is required for its functioning [13,14,15,16]. Fe–S metabolism may also play critical roles in the maintenance of genomic stability as several critical DNA metabolic enzymes require interacting with Fe–S clusters for proper function, including all DNA polymerases and several DNA helicases (e.g., DNA primase, XPD, and FancJ) [17,18,19,20]. For example, DNA polymerase ε contains a [4Fe-4S] cluster within its catalytic subunit [21]. 

**Figure 1:**
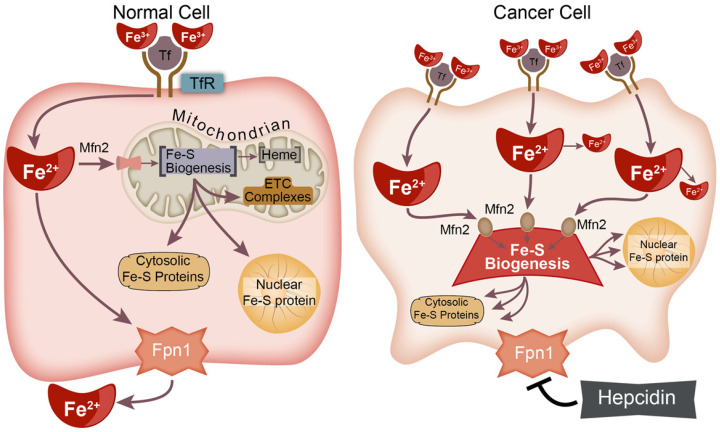
Fe–S biogenesis is theorized to represent a fundamental difference between cancer and non-malignant cells. Cancer cells frequently exhibit a phenotype characterized by preferential Fe uptake and downregulation of Fe export. The underlying mechanism is unclear, but we hypothesize that this may be related to increased cancer cell needs for Fe–S biogenesis to produce cytosolic and nuclear Fe–S proteins necessary for cancer cell survival.

Genomic instability is considered a primary hallmark of cancer and a driver of the neoplastic transformation process [22]. Thus, for cancer cells to persist and maintain clonogenicity, the maintenance of sub-lethal levels of genomic instability is critical. Despite the significant role of Fe complexes in functional proteins and the importance of such enzymes in cancer progression, the role of iron trafficking and the formation of these functional cofactors are often overlooked. In this manuscript, we summarize the complex Fe–S biogenesis process, provide a theoretical construct for a critical role in cancer (Figure 1), and illuminate potential strategies to target this process to enhance cancer therapy.

## 2. Overview of Fe–S Biogenesis 

Mitochondrial Fe–S biogenesis is a central feature of cellular iron metabolism. Many bacterial and mitochondrial Fe–S proteins have striking similarities, and Fe–S biogenesis is thought to be a cellular function passed down from alphaproteobacterium [23]. Common Fe–S clusters exist inside of proteins as either a [2Fe-2S]^+^, [4Fe-4S]^2+^, or [3Fe-4S]^+^ cluster [24]. The cluster type is specific to the enzyme; for example, succinate dehydrogenase (electron transport chain complex II) contains all three cluster types to facilitate electron transport [25]. The function of Fe–S clusters can be broken down into three fundamental categories: (1) electron transfer, (2) enzyme catalysis, and (3) regulation of biological processes [24]. Because Fe–S clusters often play a critical role in enzymatic processes, protein function is therefore largely dependent on Fe–S cluster formation via the mitochondrial biogenesis process.

Fe–S cluster biogenesis is a complex, stepwise process by which Fe–S clusters are formed in the mitochondria, exported into the cytosol, and inserted into the appropriate protein. At the basic level, the process of mammalian biogenesis occurs in four steps [1,12]:

1. [2Fe-2S] cluster formation on a scaffold protein ISCU;
2. Chaperone protein-mediated release and trafficking of the newly formed [2Fe-2S] cluster to target proteins;
3. Conversion of [2Fe-2S] clusters to [4Fe-4S] clusters;
4. Incorporation of newly formed [4Fe-4S] clusters into apo-proteins.

Each of these processes has been more extensively studied in bacteria (e.g., [2Fe-2S] cluster formation on ISCU), but they are currently being investigated actively in mammalian systems. For our purposes, we will be referring to these pathways using the human acronyms according to the HUGO Gene Nomenclature Committee. 

### 2.1. Step 1: [2Fe-2S] Synthesis on ISCU 

In prokaryotic systems, the formation of [2Fe-2S] clusters on IscU is mediated by IscS. IscU is a scaffold protein that provides an assembly platform for cluster formation with a transient Fe–S docking site [12]. The cysteine desulfurase, IscS, forms a complex with IscU to enhance [2Fe-2S] cluster formation. The crystal structure of IscU–IscS reveals that Cys-63 binds to IscS near a flexible cysteine (Cys328) loop, allowing for disulfide bridge formation [26]. IscS binds to IscU with high affinity and can donate the initial sulfur to begin [2Fe-2S] formation [27]. IscU exists in two interconverting isoforms, structured (S) and disordered (D). At pH = 8.0 and 25 °C, nearly 70% of IscU exists in the S–state that can be stabilized by the binding of Fe^2+^ or Zn^2+^ [28]. IscS preferentially binds D-IscU (*K_D_* = 2 μM) and increases the rate of S→D conversion [27]. In vitro cluster formation experiments reveal that S ↔ D equilibrium is of functional importance for Fe–S assembly [28]. IscU variants favoring the S-state showed a lag phase for cluster formation, while variants favoring the D-state did not. Despite immediate cluster formation, variants favoring the D-state formed less-stable clusters. Therefore, IscU likely exists in a D-state for high-affinity IscS binding and converts to an S-state following cluster formation for stabilization. 

**Figure 2:**
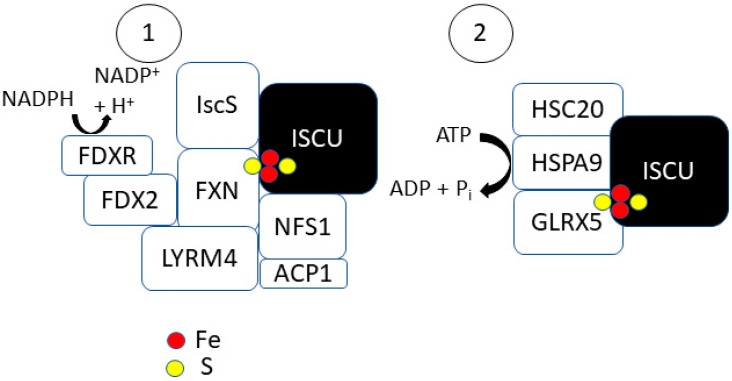
Factors involved in [2Fe-2S] cluster formation and transfer by the scaffold protein, ISCU. (1). Initial cluster formation occurs on ISCU with sulfur donation occurring by the cysteine desulfurases, NFS1/IscS (in prokaryotes). Frataxin binds at the interface of ISCU and NFS1 to allow for access to the PLP site to allow for persulfide transfer to ISCU. FXN facilitates this process. LYRM4 and ACP1 enhanceS complex stability. (2). Following completion of the [2Fe-2S] cluster on ISCU, an ISCU[2Fe-2S]-HSC20-HSPA9 complex is formed that facilitates the ATPase activity of HSPA9 allowing for [2Fe-2S] transfer to GLRX5. GLRX5 can rapidly transfer the [2Fe-2S] cluster to target proteins.

In eukaryotic systems, [2Fe-2S] cluster formation on ISCU is mediated by an NFS1 complex (Figure 2) [12]. NFS1 is a cysteine desulfurase that is accompanied by companion proteins LYRM4 and ACP1. The NFS1–LYRM4–ACP1 complex allows for the efficient formation of transient persulfides (-SSH) on cysteine residues at the active site of Nfs1 for sulfur donation. NFS1 acts as a homodimer and thus provides two independent binding sites for ISCU. LYRM4 belongs to the leucine–tyrosine–arginine motif (LYRM) family of enzymes that can bind to electron transport chain complexes I–III and V [29]. A homozygous mutation in LYRM4 results in the decreased activity of the three Fe–S containing electron transport chain complexes (I, II, III) [30]. LYRM4 is thought to enhance the stability of the ISCU-NFS1 complex [31]. LYRM4 deletions in yeast cells significantly decrease Nfs1 desulfurase activity [12]. LYRM4 can bind with ACP1 on the ISCU scaffold to form a [NFS1]_2_:[LYRM4]_2_:[ACP1]_2_ complex [32]. ACP1 is an acyl-carrier protein that provides a covalently bound 4′-phosphopantetheine (4-PP)-conjugated acyl chain to support optimal NFS1 desulfurase activity [33]. ACP1 or LYRM4 are considered essential to the early acting complex as depletion has been shown to result in defective Fe–S biogenesis [31,33,34,35].

Following the ISCU-NFS1 binding, persulfide transfer from NFS1 to ISCU is regulated by frataxin (FXN) [36,37,38]. Using cryo-electron microscopy, FXN has been shown to bind at the interface of NFS1 and ISCU to stabilize key loop confirmations of NFS1 and ISCU at this interface [39]. The NFS1 desulfurase reaction occurs at the NFS1–ISCU interface on a pyridoxal 5′-phosphate (PLP) cofactor [12]. The PLP can form a Schiff base with a free L-cysteine to allow for sulfur release. By stabilizing the interface and inducing a conformational change in NFS1, FXN allosterically regulates the desulfurase reaction by controlling access to the PLP site [38]. LYRM4 deficiency results in symptoms caused by frataxin deficiency associated with Friedreich’s ataxia, leading to the conclusion that LYRM4 facilitates FXN regulation of [2Fe-2S] cluster formation [40]. This suggests that the FXN–NFS1–ISCU complex formation occurs through LYRM4. The formation of the ISCU–NFS1–LYRM4–FXN complex and the notion that FXN interacts with ISCU, NFS1, and LYRM4 is also supported by co-immunoprecipitation data in human (HeLa) cells [41]. Once ISCU gains access to the PLP site, a cysteine residue in its active site removes a sulfur atom from the Schiff base to form a persulfide that is shuttled to the active site of ISCU [12,42]. 

Biochemically, Fxn is suggested to also aid in the formation of [2Fe-2S] by stabilizing the quaternary complex of ISCU–NFS1–LYRM4, regulating desulfurase activity, and also delivering Fe^2+^ to the complex [43,44]. Fxn can bind up to seven iron atoms [45,46]. Fxn has a higher binding affinity for ferrous iron (*K_D_*(Fe^2+^) ≈ 55 μM) compared to ferric iron (*K_D_* ≈ 11.7 μM) [45]. Fe–S reconstitution assays reveal that native, Fe–bound FXN is capable of binding to ISCU and reconstitutes a [2Fe-2S] cluster on ISCU with a *k* = 0.126 min^−1^ [46]. Using holo-FXN for iron delivery in combination with inorganic sulfide or NFS1 for sulfur delivery allowed for reconstitution of a [2Fe-2S] cluster on IscU (*k_obs_* ≈ 0.075 min^−1^) [45]. These reconstitution experiments were conducted with DTT, indicating a necessary reduction step. The in vitro reduction occurs via the [2Fe-2S] ferredoxin, FDX2 [12]. FDX2 facilitates [2Fe-2S] cluster assembly on ISCU (*k_obs_* = 0.05 s^−1^) with a rate that is slower than DTT (*k_obs_* = 0.16 s^−1^) [47]. In the mitochondria, ferredoxin-NADP^+^ reductase (FDXR) oxidizes NADPH allowing electrons to be transferred to FDX2 Equation (1) to be used for [2Fe-2S]^2+^ formation on ISCU [12].

(1) $$2{\;\mathrm{FDX}}_{\mathrm{ox}}+\mathrm{NADPH}\;\overset{\mathrm{FDXR}}{↔}\;2{\;\mathrm{FDX}}_{\mathrm{red}}+{\mathrm{NADP}}^{+}+{\;H}^{+}$$

Only the reduced form of FDX2 has been shown to bind with IscU, and it is suggested that the transfer of electrons from FDX2 to ISCU facilitates its release from the complex [48]. Due to its ability to bind ISCU and transfer an electron to the complex, FDX2 (in combination with L-cysteine) facilitates the transfer of Fe^2+^ from Fe–bound FXN to the ISCU–NFS1–LYRM4–ACP1 complex [44]. More recently, it has been shown that Fe^2+^ can bind to ISCU independent of FXN, driving persulfide uptake from NFS1 [49]. In this study by Gervason et al., FXN was shown to enhance [2Fe-2S] reconstitution by accelerating persulfide transfer and not transferring Fe. This refutes the notion of FXN as the Fe-delivery mechanism of [2Fe-2S] cluster formation on ISCU. Therefore, the chemical formation of a [2Fe-2S] cluster can be described in the following equation Equation (2):

(2) $$\mathrm{apo}-\mathrm{IscU}+2{\mathrm{Fe}}^{2+}+2S^{-}+2e^{-}\;\overset{\mathrm{FXN}}{\to }\mathrm{IscU}{\left[2\mathrm{Fe}-2S\right]}^{2+}$$

where S^−^ may be provided by the cysteine desulfurases NFS1/IscS and the complex is stabilized by LYRM4/ACP1, while FDX2 provides the two necessary electrons. 

### 2.2. Step 2: [2Fe-2S] Trafficking 

Following the formation of a [2Fe-2S] cluster, it is released from IscU and transferred to the monothiol glutaredoxin (GLRX5) (Figure 2) [12]. The transfer is facilitated by Hsp70 chaperone proteins, HSPA9 and HSC20 [50]. Much of the work completed in studying the role of chaperone proteins in [2Fe-2S] trafficking was performed in prokaryotic systems; similar functions for the human chaperone proteins have yet to be identified in vitro. The completion of the [2Fe-2S] cluster on ISCU may result in an S-state conversion that destabilizes the IscU–IscS complex and promotes IscS dissociation [28]. HSC20 (HscB in bacteria) can then recognize the IscU complex with a completed [2Fe-2S] cluster and facilitate its transfer to HSPA9 (HscA in bacteria) [51,52]. HSC20 has both ISCU and IscS binding sites at its C-terminal domain and an HSPA9 binding site at its N-terminal J-domain [53,54]. HSPA9 binding to HSC20 increases HSPA9′s ATPase activity, suggesting that HSC20 may act as a catalyst in this process [55]. HSPA9 exists in a low-peptide affinity (T-state) or high-peptide affinity (R-state). Both HSC20 and ISCU interact with HSPA9 in its T-state to enhance R-state conversion (60-fold) and rate of ATP hydrolysis (500-fold). Following the ADP/ATP exchange on HSPA9, ISCU can also stimulate R-T state conversion to restore the low-affinity state of HSPA9, allowing for ISCU dissociation [56]. The nucleotide exchange factor Mge1 assists in the dissociation of ADP from HSPA9 to allow for ATP hydrolysis [57]. Bonomi et al. showed that the catalysis of HSPA9-mediated [2Fe-2S] transfer has an absolute dependence on both HSC20 and ATP in bacteria [58]. They showed evidence of the formation of an HSPA9–HSC20–IscU_2_ complex with 1:1:1 stoichiometry with a single ATP hydrolysis step to accelerate the [2Fe-2S] transfer involving a structural change in IscU. The energy released from the ATP hydrolysis on HSPA9 allows for the release of the [2Fe-2S] from IscU and transfer to GLRX5 [57]. GLRX5 binds to HSPA9 at a non-substrate binding site to allow for cluster transfer but does not affect the ATPase activity of HSPA9 [57]. In yeast, GLRX5 depletion revealed a significant increase in the amount of [2Fe-2S] clusters bound to ISCU [59]. The transfer of the [2Fe-2S] cluster from GLXR5 occurs rapidly without the aid of any additional factors [60]. GLRX5 may be a critical connection in overall iron homeostasis as yeast cells with GLRX5 depletion have significant iron accumulation and inactivation of Fe–S-containing enzymes [61]. 

### 2.3. Steps 3 and 4: [4Fe-4S] Formation and Trafficking to Apo-Proteins

**Figure 3:**
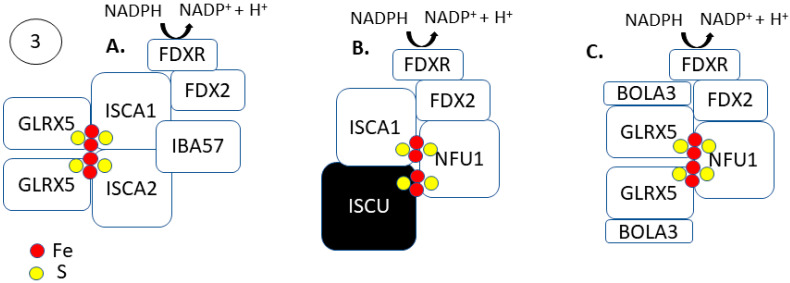
Step 3: Factors involved in mitochondrial [4Fe-4S] cluster formation. (**A**) Following [2Fe-2S] cluster formation and transfer to GLRX5, 2 molecules of holo-GLRX5 can bind with ISCA1/2. The formation of a heterodimer of [2Fe-2S]-ISCA1/2 allows for IBA57 and FDX2 dependent [4Fe-4S] assembly. (**B**) Following [2Fe-2S] cluster formation on ISCU, holo-ISCU and holo-ISCA1 can bind at the C-terminal domain of NFU1 to allow for [4Fe-4S] cluster formation on NFU1 in an FDX2-dependent manner. (**C**) Following the transfer of a [2Fe-2S] cluster from ISCU to GLXR5, a holo-GLXR5-BOLA3 complex can donate a [2Fe-2S] cluster to NFU1 for [4Fe-4S] cluster assembly. This is reduction-dependent and likely dependent on FDX2.

The formation of [4Fe-4S] clusters occur via an independent, late-acting complex of ISCA1–ISCA2–IBA57 following [2Fe-2S] cluster synthesis and trafficking through GLRX5 (Figure 3) [60]. The late-acting complex of ISA1–ISA2–IBA57 does not interact with the early acting [2Fe-2S] synthesis machinery but is dependent on the delivery of the cluster from GLRX5 [61,62,63,64]. The late-acting complex then utilizes the [2Fe-2S] clusters donated from GLRX5 for [4Fe-4S] formation. Two molecules of GLRX5-[2Fe-2S] donate their respective clusters to a heterodimeric apo-ISCA1/2 complex for [4Fe-4S] cluster formation [65]. The ISCA1/2 heterodimer helps assemble the [4Fe-4S] cluster following a donation from GLRX5. Weiler et al. showed that FDX2 catalyzes the reductive [2Fe-2S]^2+^ cluster fusion on ISCA1-ISCA2, and it is dependent on IBA57 to form [4Fe-4S]^2+^ clusters [63]. Like the early acting [2Fe-2S] machinery, the synthesis of [4Fe-4S] clusters is also dependent on the mitochondrial ferredoxin FDX2 and its associated NADPH-coupled reductase FDXR.

Following the formation of a [4Fe-4S] cluster, intra- and extramitochondrial trafficking to the appropriate apo-proteins occur. Like ISCA1/2, the mitochondrial [4Fe-4S] cluster protein, NFU1, helps assemble and facilitate intramitochondrial [4Fe-4S] trafficking. The [4Fe-4S]^2+^ ISCA1/2 heterodimer can directly transfer the cluster to NFU1 (Figure 3A) [66]. Holo-ISCA1 and holo-ISCU can also bind directly to NFU1 to donate a [2Fe-2S] cluster for [4Fe-4S] cluster formation in an FDX2 dependent manner (Figure 3B) [67]. The Fe–S cluster binding region of ISCA1/ISCU is on two α-helices in the C-terminal domain of NFU1 (*K_D_* = 1.1 μM) [67,68]. Holo-GLRX5 is also able to facilitate [4Fe-4S] cluster formation on NFU1, donating a [2Fe-2S] cluster (Figure 3C) [69]. The GLRX5 mediated [4Fe-4S] cluster formation on NFU1 requires the initial formation of a GLRX5–BOLA3 complex and a reduction step, possibly FDX2 [70]. Following [4Fe-4S] cluster formation and transfer, Nfu1 is thought to directly donate its [4Fe-4S] cluster to mitochondrial apo-proteins. Mutations in the ISCU/ISCA1 binding region of Nfu1 in patient-derived fibroblasts lost their ability to form a [4Fe-4S] cluster, resulting in decreased biochemical function of several [4Fe-4S] proteins, including lipoic acid synthase and succinate dehydrogenase [67]. These data support the notion that NFU1 aids in trafficking [4Fe-4S] clusters to target proteins.

**Figure 4:**
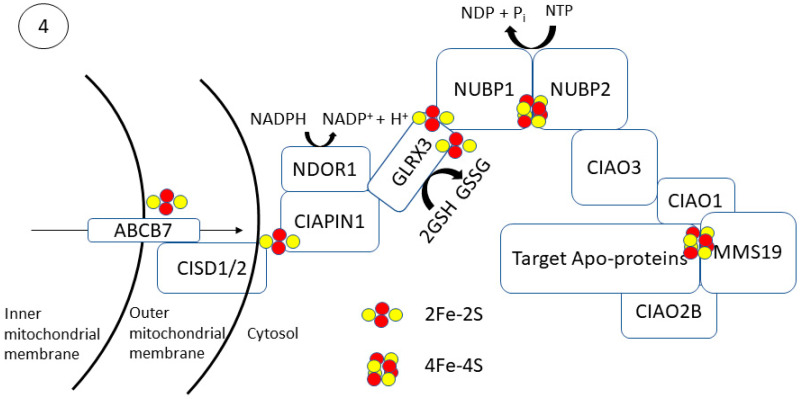
Formation and trafficking of [4Fe-4S] clusters to extramitochondrial target proteins occur via the cytosolic iron–sulfur assembly (CIA) pathway. The transmembrane protein, ABCB7, transfers the appropriate Fe and S for de novo extramitochondrial cluster synthesis, thought to be in the form of a glutathione-coordinated [2Fe-2S] cluster ([2Fe-2S](SG)_4_). On the outer mitochondrial membrane, CISD1/2 are thought to be able to transfer a reduced [2Fe-2S] cluster to the CIAPIN-NDOR1 complex, facilitated by GLRX3. This [2Fe-2S] cluster may be transferred for *de novo* [4Fe-4S] synthesis on the NUBP1/2 scaffolding complex facilitated by CIAO3. The [4Fe-4S] cluster is transferred to the CIAO1–CIAO2B–MMS19 complex, where MMS19 can directly interact with cytosolic and nuclear target apo-proteins for cluster insertion.

Trafficking of [4Fe-4S] to cytosolic and nuclear apo-proteins occurs via the cytosolic iron–sulfur assembly (CIA) pathway (Figure 4). This process begins with the transfer of Fe and S through the transmembrane protein, ABCB7 [70]. Studies in yeast, zebrafish, mice, and human cells have shown that ABCB7 deletion has a minimal effect on mitochondrial [2Fe-2S] proteins but results in functionally deficient cytosolic and nuclear [4Fe-4S] proteins [71,72,73]. A glutathione-coordinated [2Fe-2S] cluster ([2Fe-2S](SG)_4_) has been proposed as a natural substrate to be transported through ABCB7 and activate its ATPase activity (*K_D_ =* 68 μM) [74,75]. Following transport of the ([2Fe-2S](SG)_4_) across the inner mitochondrial membrane by ABCB7, it allows for cytosolic [4Fe-4S] cluster biogenesis.

Cytosolic [4Fe-4S] cluster biogenesis in eukaryotes is a complex process requiring several enzymes and has not been fully resolved. The process is thought to begin with the NEET proteins on the outer mitochondrial membrane followed by [2Fe-2S] cluster transfer via CIAPIN1 to the CIA assembly factors for MMS19-mediated insertion into target apo-proteins (Figure 4). MitoNEET (CISD1) and NAF-1 (CISD2) are [2Fe-2S] proteins located on the outer membrane of the mitochondria that aid in the export and completion of extramitochondrial Fe–S proteins [76]. Both CISD1 and CISD2 are presumed to transfer their [2Fe-2S] cluster to anamorsin (CIAPIN1); however, their function has not been elucidated definitively [77]. The CIAPIN1/NDOR1 complex directly interacts with mitoNEET (CISD1) to reduce the [2Fe-2S] cluster [78]. NDOR1 is a flavoprotein that forms a complex with CIAPIN1 and functions to transfer an electron from NADPH to reduce the [2Fe-2S] cluster [79].

[4Fe-4S] cluster formation occurs on the NUBP1–NUBP2 scaffold complex [80,81,82,83]. The [2Fe-2S] cluster of the CIAPIN1/NDOR1 complex is transferred to the NUBP1–NUBP2 via GLRX3 [81]. GLRX3-[2Fe-2S]_2_ can directly interact with the N-terminal domain of NUBP1 to transfer its cluster and facilitate [4Fe-4S] cluster formation in a glutathione-dependent manner. NUBP1 is a soluble P-loop NTPase that has four conserved cysteine restudies to carry an Fe–S cluster [82]. NUBP1 and NUBP2 contain conserved cysteine residues with a CPXC motif at their C-terminal domain coordinate a bridging [4Fe-4S] cluster [84]. In yeast, mutations of the CPXC resulted in a decrease in protein function and cell viability due to an inability to coordinate the [4Fe-4S] cluster. Mutation of the nucleotide-binding motif of NUBP1 prevented Fe–S cluster formation, suggesting that cluster fordmation and loading is a nucleotide-dependent process. The [4Fe-4S] cluster formed on the NUBP1–NUBP2 complex is also dependent on CIAO3 binding [85]. CIAO3 has conserved cysteine motifs at its N- and C-terminal domains for Fe–S binding [86]. Mutation of the Fe–S coordinating cysteine residues results in functionally deficient CIAO3, leading to a decrease in cell viability. CIAO3 knockdown in HeLa and Hep3B cells resulted in a decrease in cytosolic aconitase activity but did not affect mitochondrial aconitase activity [87]. Gari et al. initially showed that MMS19 forms a complex with CIOA1, CIAO2B, AND CIAO3 [88]. CIAO3 transfers the cluster to the CIA targeting complex consisting of CIAO1, CIAO2B, and MMS19 by acting as an external component of the complex [89]. CIAO2B and CIAO1 associate with the C-terminus of MMS19 to form a docking site for target proteins, although the XPD helicase can interact with MMS19 independent of CIAO2B/CIAO1 [90]. There appears to be a regulatory feedback mechanism for this complex as MMS19 binding prevents CIAO2B proteasomal degradation [91]. MMS19 can then transfer the [4Fe-4S] cluster to target proteins. In the 2012 study by Gari et al., it was shown that MMS19 also interacts with DNA metabolic proteins necessary for maintaining genomic stability [88]. MMS19 knockdown showed a decrease in expression of Fe–S containing DNA helicases XPD and FancJ and DNA polymerase. More recently, MMS19 depletion in HeLa cells resulted in decreased enzymatic activity of the Fe–S protein dihydropyridine dehydrogenase (DPYD) and strongly decreased levels of the POLD1 subunit of DNA polymerase δ [92].

The CIA pathway may be critical in cell survival as several DNA metabolic enzymes are dependent on complete [4Fe-4S] clusters for activity. In the 2012 study by Gari et al., it was shown that MMS19 also interacts with DNA metabolic proteins necessary for maintaining genomic stability [88]. MMS19 knockdown showed a decrease in expression of Fe–S containing DNA helicases XPD and FancJ and DNA polymerase. More recently, MMS19 depletion in HeLa cells resulted in decreased enzymatic activity of the Fe–S protein dihydropyridine dehydrogenase (DPYD) and strongly decreased levels of the POLD1 subunit of DNA polymerase δ [92]. These findings underscore the critical nature of efficient Fe–S biogenesis for cell survival. Of particular interest is the requirement of Fe–S cluster containing proteins to maintain genomic stability, a critical hallmark of cancer. Several DNA metabolic enzymes involved in DNA replication, DNA synthesis, and DNA repair, including polymerases, primase, helicases, nucleases, glycosylases and demethylases, and ribonucleotide reductases, require Fe–S biogenesis for enzymatic function and are summarized in detail by Puig et al. [93]. Currently, the critical biological role of Fe in DNA metabolism remains unknown, but emerging data are suggestive that the CIA pathway and Fe–S biogenesis is essential in maintaining genomic stability.

## 3. Fe–S Biogenesis and Cancer Initiation

The role of Fe–S biogenesis in carcinogenesis is currently unclear. However, according to the TNMplot database, several Fe–S biogenesis genes are altered in tumor tissue compared to their normal tissue counterparts [94]. The TNMplot database revealed that several genes involved in [2Fe-2S] synthesis and trafficking are overexpressed in a variety of cancer types as compared to normal tissues (Table 1). While the majority of [2Fe-2S] synthesis appears to be upregulated, ISCU may be downregulated. This is not surprising as ISCU is positively regulated by p53 through an intronic binding site and was decreased in human liver cancer tissues [95]. Additionally, ISCU has been shown to be downregulated in cancers by the hypoxia-inducible-factor (HIF)-mediated miR-210, leading to decreased ETC complex I and aconitase activity while enhancing cancer cell survival [96]. In this study, ISCU suppression associated with miR-210 showed a worse clinical prognosis in breast cancer and head and neck cancer patients.

Due to its well-known role in Friedreich’s Ataxia (FA) [97], FXN has been a point of interest in cancer research. There are limited data regarding the probability of cancer incidence in individuals diagnosed with FA, although case studies have reported gastric carcinoma and breast cancer development in siblings with FA [98,99]. The limited data regarding FA and cancer development may be related to lifespan, as the average life expectancy of FA patients was reported to be 36.5 years while the average age of cancer diagnosis is between 65 and 70 years [100,101]. In yeast cells, the absence of FXN (Yfh1 in yeast) was shown to lead to nuclear damage resulting from Fe-catalyzed reactive oxygen species formation [102]. FXN overexpression has been shown to promote DNA repair via base excision repair in mammalian hamster fibroblasts [103]. FA mouse fibroblast cells have much higher levels of genomic instability, and wild-type FXN gene transfer has been shown to reverse the impaired DNA repair associated with FA [104]. These findings illustrate how early acting Fe–S biogenesis may be essential for cancer cells to maintain the adequate genomic stability required for disease progression and tumor formation. FXN was first shown to be overexpressed in cancer cells in 2001, where Serio et al. showed that FXN participates in hypoxia-induced stress responses in tumors through a HIF mechanism [105]. The HIF-induced overexpression of FXN is similar to the associated downregulation of ISCU [96]. It was also previously shown that disruption of FXN expression in mouse hepatocytes resulted in impaired mitochondrial respiration, increased oxidative stress, decreased Fe–S containing proteins, increased hepatocyte proliferation, decreased lifespan, and increased incidence of hepatocellular carcinomas [106]. Conversely, Fxn overexpression in various colon cancer cell lines has been shown to have increased oxidative metabolism and aconitase activity while mitigating tumor cell growth in vitro and in vivo [107]. Taken together, FXN may be thought about as an antioxidant and potential tumor-suppressor but its role in the various stages of cancer development remain unclear. In addition to FXN, NFS-1 suppression in lung cancer cells results in reduced cell growth, impaired cysteine transport, and the induction of ferroptosis in vitro [108]. In an in vivo mouse model, NFS-1 suppression resulted in the inability to form both primary lung adenocarcinomas and metastatic lung tumors.

Along with [2Fe-2S] synthesis, the trafficking of Fe–S clusters may also be important for cancer cell survival. The silencing of GLRX5 in head and neck cancer cells can reverse cisplatin resistance and enhance the induction of ferroptosis both in vitro and in vivo [109]. The Fe–S chaperone, HSPA9, may play a role in carcinogenesis and be a therapeutic target as it negatively regulates the oncogenic Raf/MEK/ERK pathway [110]. Similarly, HSPA9 inhibition enhanced cell death in KRAS-mutant pancreas tumors [111]. Taken together, these data support the notion that the aberrant activity of the early acting [2Fe-2S] pathway may be oncogenic and a novel therapeutic target.

In addition to evaluating the early acting [2Fe-2S] machinery, recent studies have evaluated [4Fe-4S] trafficking proteins and their roles in cancer. TNMplot analysis reveals overexpression of several proteins involved in [4Fe-4S] trafficking from the mitochondria and to the cytosol and nucleus (Table 2). The NEET family of proteins (CISD1/2) at the beginning of the CIA pathway is thought to be a novel cancer target [112]. CISD2 overexpression in murine xenograft breast cancer tumors resulted in increases in tumor size, aggressiveness, and the tolerance of cells to oxidative stress [113]. In this study, a point mutation was made in CISD2 (H114C) that stabilized the 3Cys–1His [2Fe-2S] cluster of NAF-1 to prevent cluster transfer. Remarkably, this lone point mutation resulted in a significant decrease in tumor size and tolerance to oxidative stress. This finding illuminates how enhanced trafficking of Fe–S clusters to target apo-proteins may be critical for tumor progression and survival. The development of a mitochondrially targeted therapeutic (MAD-28) has been shown to selectively kill breast cancer cells (MDA-MB-231) in vitro with no significant cytotoxicity on normal breast epithelial cells [114]. MAD-28 forms an H-bonding network inside of CISD1/2 to stabilize the [2Fe-2S] cluster and prevent transfer down the CIA pathway. MMS19, the terminal enzyme of the CIA pathway, has also been investigated as a potential cancer target. By altering DNA metabolic enzyme stability, MMS19 depletion in HeLa cells resulted in significant sensitivity to DNA damage and genomic instability [92]. MMS19 is tagged for proteasomal degradation by the ubiquitin ligase, MAGE-F1, in lung cancer [114]. While MAGE-F1 overexpression enhanced tumor growth, the associated degradation of MMS19 decreased DNA repair capacity and sensitized cells to ultraviolet radiation-induced DNA damage in HEK293 cells. These data suggest two critical points: (1) significant work is required for deconvoluting the intricate role of Fe–S biogenesis in tumor growth and (2) Fe–S cluster trafficking pathways and [4Fe-4S]-containing proteins may be critical targets for modulating tumor sensitivity to therapy. The upregulation of the CIA pathway may be critical for tumors as they aim to maintain clonogenicity and progress. This pathway may be critical for tumors considering several nuclear proteins involved in maintaining genomic stability require Fe–S clusters [17]. However, more mechanistic data are required to fully elucidate the role of the CIA pathway and associated Fe–S containing enzymes (e.g., DNA polymerases) in the development of cancer.

## 4. Potential Therapeutic Strategies to Target Fe–S Clusters in Cancer

**Figure 5:**
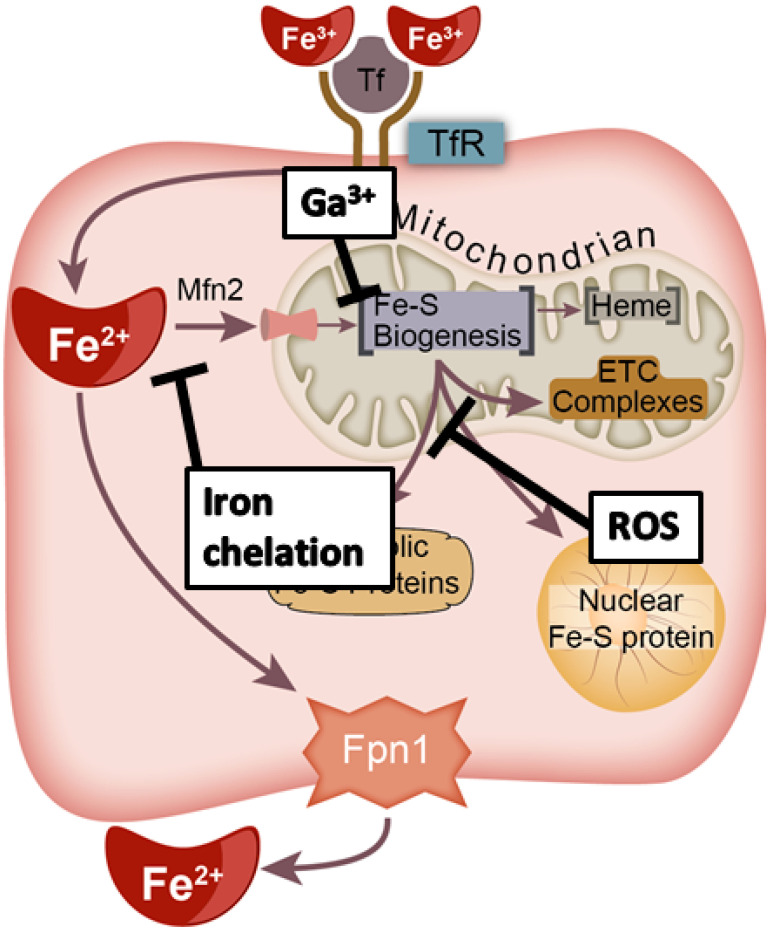
Therapeutic strategies to target the Fe–S biogenesis pathway. Current strategies to target the Fe–S biogenesis pathway to induce cancer cell killing include Fe chelation to prevent intracellular trafficking, iron mimicry in the form of Ga^3+^ to function as a Fe antagonist and impair the Fe–S biogenesis process, and pro-oxidants (e.g., ionizing radiation) to perturb Fe–S containing proteins.

Because Fe–S metabolism is theorized to play a critical role in cancer development, progression, and/or therapeutic resistance, targeting this complicated network with compounds currently under clinical investigation may provide a novel approach to selectively disrupt cancer cell metabolism due to fundamental differences between cancer and non-malignant cells [115,116,117]. There are several possible methods to disrupt the Fe–S metabolic network at different levels (Figure 5): (i) redox manipulations that disrupt Fe–S cluster stability, (ii) iron chelation to limit the iron availability of the cell to prevent cluster formation, and (iii) replacement of iron with redox inactive metals to halt Fe–S biogenesis and function of Fe–S containing proteins.

### 4.1. Redox Manipulation

**Figure 6:**
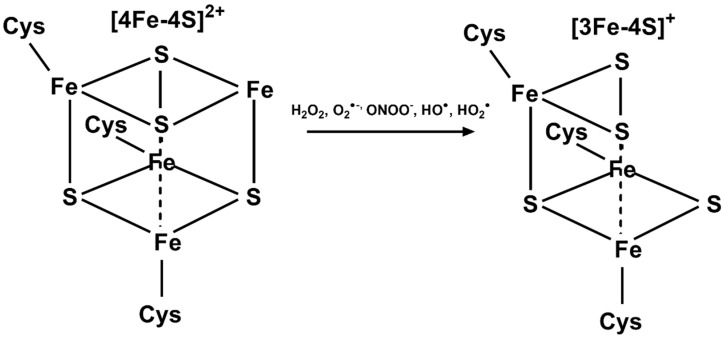
ROS-mediated oxidation of aconitase-like [4Fe-4S] clusters. ROS (H_2_O_2_, O_2_^•−^, ONOO^−^, HO^•^, HO_2_^•^) can oxidize the aconitase [4Fe-4S]^2+^ cluster leading to the release of a non-cys bound Fe atom.

Once completed and adequately formed, Fe–S clusters can be chemically altered by reactive oxygen species (ROS). As previously described, Fe–S proteins contain either a [2Fe-2S] or [4Fe-4S] cluster. When considering the redox chemistry of Fe–S clusters, the [4Fe-4S]^2+^ cluster of aconitase is a primary point of interest. The [4Fe-4S]^2+^ of aconitase is essential for its enzymatic activity in the conversion of citrate to isocitrate in the citric acid cycle as the solvent-exposed Fe site is a Lewis acid [24,118]. Increased fluxes of H_2_O_2_, O_2_^•−^, ONOO^−^, and HO_2_^•^ can oxidize the [4Fe-4S]^2+^ cluster (Figure 6) [119]. The oxidation of the cluster leads to a subsequent release of the solvent-exposed iron atom and enzyme inactivation [118,119,120]. Thus, aconitase is left in its inactive state with an oxidized [3Fe-4S]^+^ cluster. In this regard, aconitase activity is considered a useful marker of intracellular ROS stress. Along with enzyme inactivation, the production of a [3Fe-4S]^+^ cluster is accompanied by the release of a redox-active Fe atom that can go on to further catalyze oxidation reactions and cause further cellular damage.

One promising chemical therapy to alter Fe–S cluster stability inside of cells is ascorbate (vitamin C; AscH^−^). AscH^−^ is classically considered an antioxidant because of its ability to act as a one-electron reductant [121]. However, AscH^−^ can readily react with catalytically active iron to selectively kill cancer cells [10]. While AscH^−^ can actively reduce Fe^3+^ to Fe^2+^ Equation (3),

(3) $${\mathrm{Fe}}^{3+}+{\;\mathrm{AscH}}^{-}\;\to {\;\mathrm{Fe}}^{2+}+{\mathrm{Asc}}^{-}$$

AscH^−^ can also generate high fluxes of intracellular H_2_O_2_ [10,122,123]. By generating increased H_2_O_2_ fluxes, AscH^−^ decreases aconitase activity, which can be reversed by catalase overexpression [10]. This is likely due to H_2_O_2_ oxidation of the non-Cys bound Fe–site of the [4Fe-4S]^2+^ cluster via Fenton chemistry Equation (4) to generate an oxidized, inactive [3Fe-4S]^+^ cluster [6].

(4) $${\;\mathrm{Fe}}^{2+}+{\;H}_{2}O_{2}\;\to {\;\mathrm{Fe}}^{3+}+{\mathrm{HO}}^{}+{\mathrm{OH}}^{-}\;$$

Supraphysiological doses of AscH^−^ (≥20 mM plasma concentrations; pharmacological ascorbate) have shown to have high clinical anticancer translational potential. Pre-clinical mouse models in lung cancer, glioblastoma, sarcoma, pancreas, and ovarian cancer have shown that high dose AscH^−^ can enhance radiation and/or chemotherapy [10,122,124,125]. Clinically, high dose AscH^−^ has significant promise as an anticancer agent when combined with standard of care cancer therapies with limited added toxicity in early phase clinical trials in brain, lung, pancreas, and ovarian cancers [10,124,125,126,127,128]. Currently, AscH^−^ is under investigation in phase II clinical trials in pancreatic cancer (NCT02905578), non-small cell lung cancer (NCT02905591), and glioblastoma tumors (NCT02344355). Additional phase I clinical trials for patients with soft tissue sarcomas (NCT04634227, NCT04877587, NCT03508726) and glioblastoma tumors (NCT04900792) are ongoing or will begin recruiting subjects soon.

Like the pro-oxidant nature of AscH^−^, ionizing radiation (IR) is also able to disrupt aconitase activity. Chinese hamster lung fibroblasts exposed to 50 cGy of γ-irradiation resulted in a >50% decrease in both cytoplasmic and mitochondrial aconitase activity [129], suggesting that ionizing radiation can chemically disrupt [4Fe-4S]^2+^ in vitro. In human lung carcinoma (A549) cells, 10 Gy of X-rays resulted in altered activity of all three Fe–S-containing mitochondrial electron transport chain complexes (I, II, and III) [130]. This suggests an IR-induced redox modulation of Fe–S cluster stability prevents electron transport chain complex activity. Currently, the intracellular redox chemistry regarding H_2_O_2_ fluxes and other cytosolic/nuclear [4Fe-4S]^2+^-containing proteins (e.g., DNA polymerases/helicases) are unclear and require further investigation. However, these data suggest that H_2_O_2_ and other ROS-generating therapies (i.e., IR) can be an efficient and translatable approach to disrupt Fe–S metabolism downstream of the Fe–S biogenesis process.

### 4.2. Iron Chelation

**Figure 7:**
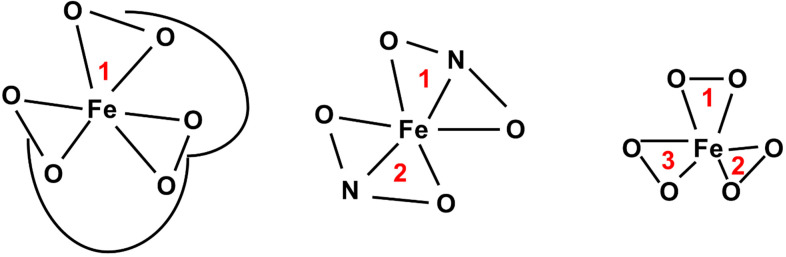
Structure of different Fe-chelator complexes. Hexadentate Fe–chelators (e.g., desferrioxamine) bind Fe with 1:1 stoichiometry. Tridentate chelators (e.g.,) bind Fe with 2:1 stoichiometry. Bidentate chelators (e.g.,) bind Fe with 3:1 stoichiometry.

Iron chelators are compounds that bind to iron with high affinity. There are several different classes of Fe chelators, but they are typically comprised of donor oxygen, nitrogen, or sulfur groups that can form up to six-coordinate bonds with iron [131]. Hexadentate chelators contain six donor atoms and can therefore bind with 1:1 stoichiometry [132]. Bidentate chelators have two donor atoms, and tridentate have three donor atoms and can bind with 3:1 and 2:1 stoichiometry, respectively (Figure 7). By taking Fe out of intracellular circulation, chelation therapy may be able to prevent Fe–S biogenesis by limiting iron and halt cellular processes that require Fe–S-containing proteins.

Desferrioxamine (DFO) is an iron chelator that forms a stable hexadentate complex with a high affinity for Fe^3+^ (*K_D_* ≈ 10^31^) [133]. DFO is the first iron chelator that was used clinically for the treatment of iron overload [134]. Due to its clinical accessibility, it was the first to be tested as an anticancer agent. Pre-clinically, DFO was shown to have anti-proliferative effects in leukemia cells both in vitro and in vivo [135]. DFO has also been shown to have dose- and time-dependent cytotoxic effects in cervical and ovarian cancer cells via a pro-apoptotic mechanism [136,137]. DFO may also enhance mitochondrial iron accumulation and ROS production in triple-negative MDA-MB-231 breast cancer cells [138]. A new, mitochondrially targeted DFO compound (mitoDFO) can suppress breast cancer tumor growth in vivo and in vitro [139]. MitoDFO can enhance cancer cell killing by impairing Fe–S biogenesis to disrupt mitochondrial function and generate ROS. Despite the interest in DFO and its pre-clinical promise as a cancer therapy, clinical trials in neuroblastoma and prostate cancer patients yielded limited benefit for DFO utilization [140,141,142]. The poor clinical trial results in combination with poor bioavailability (t_1/2, plasma_ ≈ 10 min) and subcutaneous administration of DFO have led to a search for more promising iron chelators [142].

Deferasirox (DFX) is a synthetic iron chelator that forms a tridentate complex with both Fe^2+^ and Fe^3+^ [143]. Like DFO, in vitro, DFX has dose-dependent anti-proliferative and pro-apoptotic effects in lung carcinoma, hepatoma, lymphoma, esophageal cancer, pancreas cancer, gastrointestinal cancers, and leukemia cells [144,145,146,147,148,149,150,151]. More recently, DFX has been shown to be cytotoxic to breast cancer stem cells by inhibiting the electron transport chain leading to ROS production [152]. DFX showed tumor growth inhibition in murine xenograft models of both lung and esophageal cancers [148,151]. DFX is orally available with an extended elimination half-life compared to DFO (t_1/2_ = 7–16 h), leading to potential clinical interest [153]. Taken together, these results suggest that iron chelation may warrant further investigation as a method of targeting Fe–S metabolism to enhance cancer therapies.

### 4.3. Iron Mimicry

**Figure 8:**
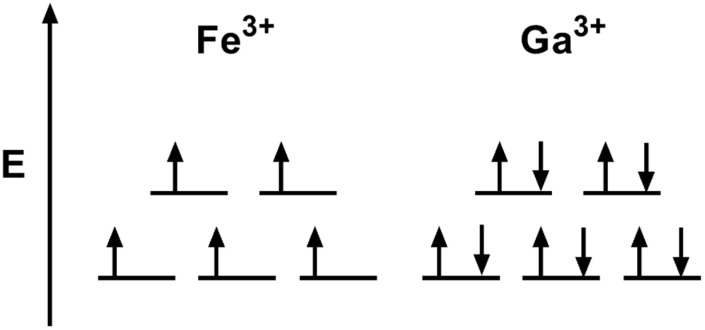
Valence electron configuration of Fe^3+^ and Ga^3+^. Energy level diagram of the d-orbital shell of Fe^3+^ and Ga^3+^ when bound in an octahedral coordination environment (6 ligands). The d-orbital of Fe^3+^ is partially filled with 5 unpaired electrons (S = 5/2) while Ga^3+^ has a d-orbital with 10 paired electrons (S = 10). The completed d-orbital of Ga^3+^ prevents redox cycling (154).

Another promising therapeutic option targeting iron metabolism is gallium (Ga). Ga is a lanthanide metal that is like iron in size and valence structure (Figure 8) [154]. Gallium (Ga^3+^) is a d^10^ metal with a full d-orbital shell (10 paired electrons, S = 0), while iron (Fe^3+^) is a d^5^ metal that has a half-filled d-orbital (5 unpaired electrons, S = 5/2). Due to its similar size and valence structure, Ga^3+^ can coordinate ligands like Fe^3+^. However, the fully paired d-orbital of Ga^3+^ likely prevents its redox cycling and transferring of electrons such as Fe [155].

Physiologically, Ga^3+^ can bind to transferrin in circulation like Fe^3+^ and be taken into cells via transferrin-receptor mediated endocytosis [156]. Once inside the cell, Ga^3+^ can act as an antagonist to divalent metal ions (e.g., Fe^2+^) [157] and also be transferred into ferritin supporting its antagonism with Fe [158,159]. The cytotoxicity of Ga^3+^ is likely related to its ability to inhibit Fe-containing proteins such as ribonucleotide reductase [155,160]. In addition, Ga^3+^ also has pro-apoptotic effects in lymphoma [161]. Moreover, Fe supplementation can reverse the cytotoxic effects of Ga^3+^ [162]. Ga^3+^-nitrate demonstrated clinical anti-cancer effects in non-Hodgkin’s lymphoma and bladder cancer and was considered well tolerated [163,164,165]. The clinical utility and tolerability of Ga^3+^-nitrate were confirmed in a multi-center, phase II clinical trial of patients with advanced non-Hodgkin’s lymphoma [166].

More recent studies have focused on the use of Ga^3+^-maltolate (GaM). GaM is an orally active compound that has greater bioavailability than Ga^3+^-nitrate [154]. The estimated half-life for GaM is 17–21 h, compared to Ga^3+^-nitrate, which has an initial half-life of 1.4 h [167,168]. Three days following an oral GaM administration, nearly all circulating Ga^3+^ was bounding to Tf, while only 2% appeared in the urine [168]. This was a significant improvement on Ga^3+^-nitrate, which had 49–94% of infused Ga^3+^ excreted within 72 h. In several lymphoma cell lines, GaM showed a significant increase in cytotoxicity when compared to Ga^3+^-nitrate [169]. In vitro, GaM can impair mitochondrial function in lymphoma cells, leading to ROS production and activation of apoptosis [169]. More recently, GaM has been shown to impair mitochondrial function by disrupting the electron transport chain and ribonucleotide reductase activity in glioblastoma cells [170]. Despite not being an Fe–S cluster, the di-Fe center in ribonucleotide reductase is also formed using the CIA machinery and is necessary for cytosolic and nuclear Fe–S maturation [171,172]. Thus, Ga^3+^ may function as an iron mimic to inhibit Fe–S biogenesis at one of the several electron transfer steps necessary for successful Fe–S formation and insertion into apo-proteins.

## 5. Conclusions

Cancer cells appear to exhibit an iron-dependent pattern of cell growth via overexpression of Fe uptake pathways and downregulation of Fe export pathways. Currently, the underlying mechanism of this phenotype remains unclear. We propose that overactivity of the Fe–S biogenesis pathway underlies the iron dependence of cancer cells by facilitating the activation of Fe–S-containing proteins necessary for tumor cell survival. Thus, the Fe–S biogenesis pathway represents a critical vulnerability in cancer cells that may be exploited via redox manipulation with pro-oxidants (e.g., AscH^−^), iron chelation (e.g., DFX), or iron mimicry (e.g., GaM). Differential iron dependence and Fe–S activity in cancer cells may theoretically present an intrinsic vulnerability to allow for normal cell protection along with cancer cell sensitization. While substantial investigation is required from both a basic biochemistry and translational sciences perspective to uncover its importance, targeting Fe–S biogenesis in cancer development and therapy presents a potential opportunity to significantly advance the field of cancer biology.

## Figures and Tables

**Table 1:** [2Fe-2S] biogenesis in tumor tissues relative to their normal tissue counterparts ^a^.

Gene	Role in Fe–S Formation	Cancer Types	Implication in Tumors ^b^
**ISCU**	[2Fe-2S] scaffold	AML, bladder, breast, colon, lung, ovary, prostate, rectum, renal (clear cell), stomach, testis, uterine	↓
		Adrenal, liver, pancreas, renal (chromophobe), thyroid	↑
**NFS1**	S donation in [2Fe-2S] synthesis	Adrenal, AML, bladder, colon, esophageal, lung, pancreas, rectum, renal (chromophobe), uterine	↑
		Renal (clear cell), testis, thyroid	↓
**FXN**	IscU scaffold stability Fe^2+^ donation	AML, breast, colon, esophageal, lung, prostate, rectum, renal (chromophobe), skin, stomach, testis, thyroid, uterine	↑
		Liver, renal (clear cell)	↓
**LYRM4**	IscU scaffold stability	AML, breast, colon, esophageal, liver, lung, ovary, pancreas, prostate, rectum, renal (papillary), skin, stomach, testis, thyroid, uterine	↑
		Renal (chromophobe)	↓
**ACP1**	IscU scaffold stability	AML, bladder, breast, colon, esophageal, liver, lung, ovary, prostate, pancreas, rectum, skin, testis, thyroid, uterine	↑
		Renal (chromophobe)	↓
**GLRX5**	[2Fe-2S] trafficking	Adrenal, breast, bladder, esophageal, liver, lung, ovary, pancreas, prostate, rectum, renal (chromophobe), skin, stomach, thyroid, uterine	↑
		AML, renal (clear cell), testis	↓
**HSC20**	[2Fe-2S] trafficking	AML, liver, lung, pancreas, prostate, renal (clear cell), skin, testis, thyroid, uterine	↑
		Adrenal, ovary, rectum, renal (chromophobe), stomach	↓
**HSPA9**	[2Fe-2S] trafficking	Adrenal, AML, breast, colon, liver, lung, pancreas, prostate, rectum, renal (chromophobe), skin, testis, uterine	↑

^a^ Gene expression data collected from tnmplot.com [94]. ^b^ Significant differences relative to associated normal cells by Mann–Whitney U test [94].

**Table 2:** [4Fe-4S] biogenesis in tumor tissues relative to their normal tissue counterparts ^a^.

Gene	Role in Fe–S Formation	Cancer Types	Implication in Tumors ^b^
**ABCB7**	Fe–S trafficking across inner mitochondrial membrane	AML, colon, esophageal, liver, lung (adenocarcinoma), pancreas, rectum, renal (clear cell and chromophobe), testis	↓
		Adrenal, breast, ovary, skin, uterine	↑
**CISD1**	Fe–S donation for extramitochondrial trafficking	Adrenal, AML, breast, colon, esophageal, liver, lung, ovary, pancreas, prostate, renal (chromophobe), skin, stomach, uterine	↑
		testis	↓
**CISD2**	Fe–S donation for extramitochondrial trafficking	Adrenal, AML, bladder, breast, colon, esophageal, liver, lung, ovary, pancreas, prostate, rectum, renal (clear cell, chromophobe, papillary), skin, stomach, testis, thyroid, uterine	↑
**CIAPIN1**	Cytosolic Fe–S transfer	Adrenal, AML, bladder, breast, colon, liver, lung, ovary, pancreas, prostate, rectum, renal (papillary), skin, stomach, uterine	↑
		Renal (clear cell), testis, thyroid	↓
**NDOR1**	Electron transfer to CIAPIN1 for *de novo* cluster transfer	AML, bladder, esophageal, ovary, stomach	↑
		Colon, lung, prostate, renal (clear cell), testis, thyroid, uterine	↓
**NUBP1**	Cytosolic [4Fe-4S] formation/transfer	Adrenal, AML, breast, colon, esophageal, liver, lung, ovary, pancreas, prostate, rectum, renal (clear cell, chromophobe, papillary), skin, stomach, testis, thyroid, uterine	↑
**NUBP2**	Cytosolic [4Fe-4S] formation/transfer	Adrenal, bladder, breast, colon, liver, lung, prostate, rectum, renal (clear cell, chromophobe, papillary), skin, testis, uterine	↑
		Stomach	↓
**CIAO3**	Cytosolic [4Fe-4S] formation/transfer	Adrenal, AML, bladder, breast, liver, lung, pancreas, rectum, renal (chromophobe, papillary), uterine	↑
		Esophageal, stomach, testis, thyroid	↓
**CIAO1**	Cytosolic [4Fe-4S] transfer	Adrenal, AML, bladder, breast, colon, esophageal, liver, lung, ovary, pancreas, prostate, rectum, renal (clear cell, chromophobe, papillary), skin, stomach, testis, thyroid, uterine	↑
**CIAO2B**	Cytosolic [4Fe-4S] transfer	AML, bladder, breast, colon, esophageal, lung, ovary, pancreas, prostate, rectum, renal (chromophobe), renal (papillary), skin, stomach, testis, thyroid, uterine	↑
**MMS19**	Insertion of [4Fe-4S] cluster into target apo-proteins	AML, liver, pancreas, renal (papillary)	↑
		Breast, lung, ovary, prostate, renal (clear cell and chromophobe), skin, testis, thyroid, uterine	↓

^a^ Gene expression data collected from tnmplot.com [94]. ^b^ Significant differences relative to associated normal cells by Mann–Whitney U test [94].

## Abbreviations

ABCB7:	ATP-binding cassette sub-family B member 7
ACP1:	Low-molecular-weight phosphotyrosine protein phosphatase
AML:	Acute myeloid leukemia
AscH^−^:	Ascorbic acid, vitamin C
BOLA3:	BolA Family Member 3
CIAO1:	Cytosolic Iron–Sulfur Assembly Component 1
CIAO2B:	Cytosolic iron–Sulfur Assembly Component 2B
CIAO3:	Cytosolic Iron–Sulfur Assembly Component 3
CIAPIN1:	Cytokine Induced Apoptosis Inhibitor 1, anamorsin
CISD1:	CDGSH Iron Sulfur Domain 1
CISD2:	CDGSH Iron Sulfur Domain 2
DFO:	Desferrioxamine
DFX:	Deferasirox
FDX1:	Ferrodoxin-1
FDX2:	Ferrodoxin-2
FDXR:	Ferrodoxin reductase
Fpn-1:	Ferroportin
FXN:	Frataxin
FancJ:	Fanconi anemia group J protein
GLRX3:	Glutaredoxin-3
GLRX5:	Glutaredoxin-5
HIF-1:	Hypoxia inducible factor-1
HSPA9:	Heat Shock Protein Family A (Hsp70) Member 9
HSC20:	HscB Mitochondrial Iron–Sulfur Cluster Cochaperone
IBA57:	Iron–Sulfur Cluster Assembly Factor IBA57
ISCA1/2:	Iron–Sulfur Cluster Assembly 1/2
ISCU:	Iron–Sulfur Cluster Assembly Enzyme
IscS:	Cysteine desulfurase (prokaryotic)
LYRM4:	LYR Motif Containing 4
Mfn2:	Mitoferrin-2
MMS19:	Cytosolic Iron–Sulfur Assembly Component
NDOR1:	NADPH-Dependent Diflavin Oxidoreductase 1
NFS1:	Cysteine Desulfurase (eukaryotic)
NFU1:	Iron–Sulfur Cluster Scaffold Protein
NUBP1/2:	Nucleotide-Binding Protein 1/2
XPD:	Xeroderma pigmentosum group D
